# Chromogranin A regulates neuroblastoma proliferation and phenotype

**DOI:** 10.1242/bio.036566

**Published:** 2019-03-15

**Authors:** Dongyun Zhang, Lilit Babayan, Hillary Ho, Anthony P. Heaney

**Affiliations:** 1 Department of Medicine, David Geffen School of Medicine, University of California, Los Angeles 90095, USA; 2 Department of Neurosurgery, David Geffen School of Medicine, University of California, Los Angeles 90095, USA

**Keywords:** Chromogranin A, Neuroblastoma, Insulin-like growth factor, Differentiation therapy

## Abstract

Neuroblastoma is a commonly encountered solid tumor in early childhood with high neuroplasticity, and differentiation therapy is hypothesized to lead to tumor mass shrinkage and/or symptom relief. CgA is a tissue specific protein restricted to the diffuse neuroendocrine system, and widely expressed in neuroblastomas. Using knockdown and knockout approaches to deplete CgA levels, we demonstrated that CgA loss inhibits SH-SY5Y cell proliferation and leads to a morphological shift with increased expression of Schwann and extracellular matrix specific molecules, and suppression of chromaffin features. We further confirmed the effects of CgA in a series of neuroblastoma cells with [BE(2)-M17 and IMR-32] and without (SK-N-SH) N-Myc amplification. We demonstrated that CgA depletion reduced IGF-II and IGFBP-2 expression, increased IGFBP-3 levels, and suppresses IGF downstream signaling as evidenced by reduced AKT/ERK pathway activation. This was further supported by an increased anti-proliferative effect of the ERK inhibitor in the CgA depleted cells. In an *in vivo* xenograft neuroblastoma model, CgA knockdown led to increased S-phenotypic marker expression at both protein and mRNA levels. Together these results suggest that CgA maintains IGF secretion and intracellular signaling to regulate proliferation and differentiation in neuroblastomas.

## Abstract (precis)

**Summary:** Loss of CgA directs neuroblastoma differentiation toward S-type, which synergize the anti-proliferative effects of AKT/MEK inhibitors, suggesting a novel treatment direction.

## INTRODUCTION

Neuroblastoma is one of the most commonly encountered early-childhood extracranial tumors, and arises from the neural crest during embryonic development. This tumor retains plasticity and can differentiate into several tissue lineages, resulting in diverse clinical manifestations in terms of lesion location, tumor composition, disease stage and progression (Ngan, 2015; Tsokos et al., 1985). Additionally, a broad range of treatment outcomes is observed, including unresponsiveness to conventional radiation and chemotherapy, treatment-induced maturation to a benign ganglioneuroma and/or ganglioneuroblastoma, and even spontaneous regression (Cooper et al., 1991).

A heterogeneous cellular composition is typically observed in neuroblastoma tumor tissues and cultured cell lines, where distinct neuroblastic (N)-, substrate-adhesive (S)- and intermediate (I)-types have been demonstrated (Ross et al., 2003). N-type cells represent immature sympathoblasts, expressing neuronal skeleton markers such as neurofilament and neurotransmitter synthesizing enzymes including tyrosine hydroxylase; S-type cells show fibroblast or epithelial-like characteristics and express smooth muscle-specific proteins, including alpha smooth muscle actin, basic calponin and desmin. The final I-type cells are considered to be stem cells that can differentiate into either N-type or S-type cells (Piacentini et al., 1996), and interconversion between N- and S-type cells has been observed (Tsokos et al., 1985). Intuitively therefore, characterization of the mechanisms underlying this plasticity and improved understanding of factors that could direct tumor differentiation toward N- or S-type could not only advance our understanding of neural crest development, but potentially provide novel therapeutic strategies for neuroblastoma disease control.

Chromogranin A (CgA, NM_001275.3) is a 456-amino acid hydrophilic acidic protein of the granin family, expressed in a variety of endocrine, neuroendocrine, peripheral and central neural tissues (Bartolomucci et al., 2011). CgA functions as a key component of dense-core secretory granules and modulates the storage and processing of neuropeptide and peptide hormones in health and disease (Helle, 2004). Circulating CgA levels are elevated in a variety of neuroendocrine tumors (NETs), including carcinoids, pancreatic NETs, pheochromocytoma, paraganglioma, and neuroblastoma (Modlin et al., 2010). Serum CgA levels in neuroblastoma patients correlate with tumor burden and can be used as a sensitive and specific diagnostic and prognostic disease marker (Hsiao et al., 1990; Pagani et al., 2002). *In vitro* studies have demonstrated alterations in CgA transcription during neuroblastoma differentiation induced by retinoic acid and cAMP (Gaetano et al., 1995). However, the potential role, if any, for CgA itself in regulating neuroblastoma proliferation and/or differentiation remains unclear. In the current study, we have characterized CgA effects in a series of neuroblastoma cell lines and demonstrated that CgA depletion results in reduced neuroblastoma proliferation *in vitro* and *in vivo* and changes the neuroblastoma phenotype, indicating that CgA may be a promising therapeutic target for treatment of neuroblastoma and potentially other neuroendocrine tumors.

## RESULTS

### shRNA-directed CgA depletion inhibits *in vitro* neuroblastoma cell proliferation

**Fig. 1.**
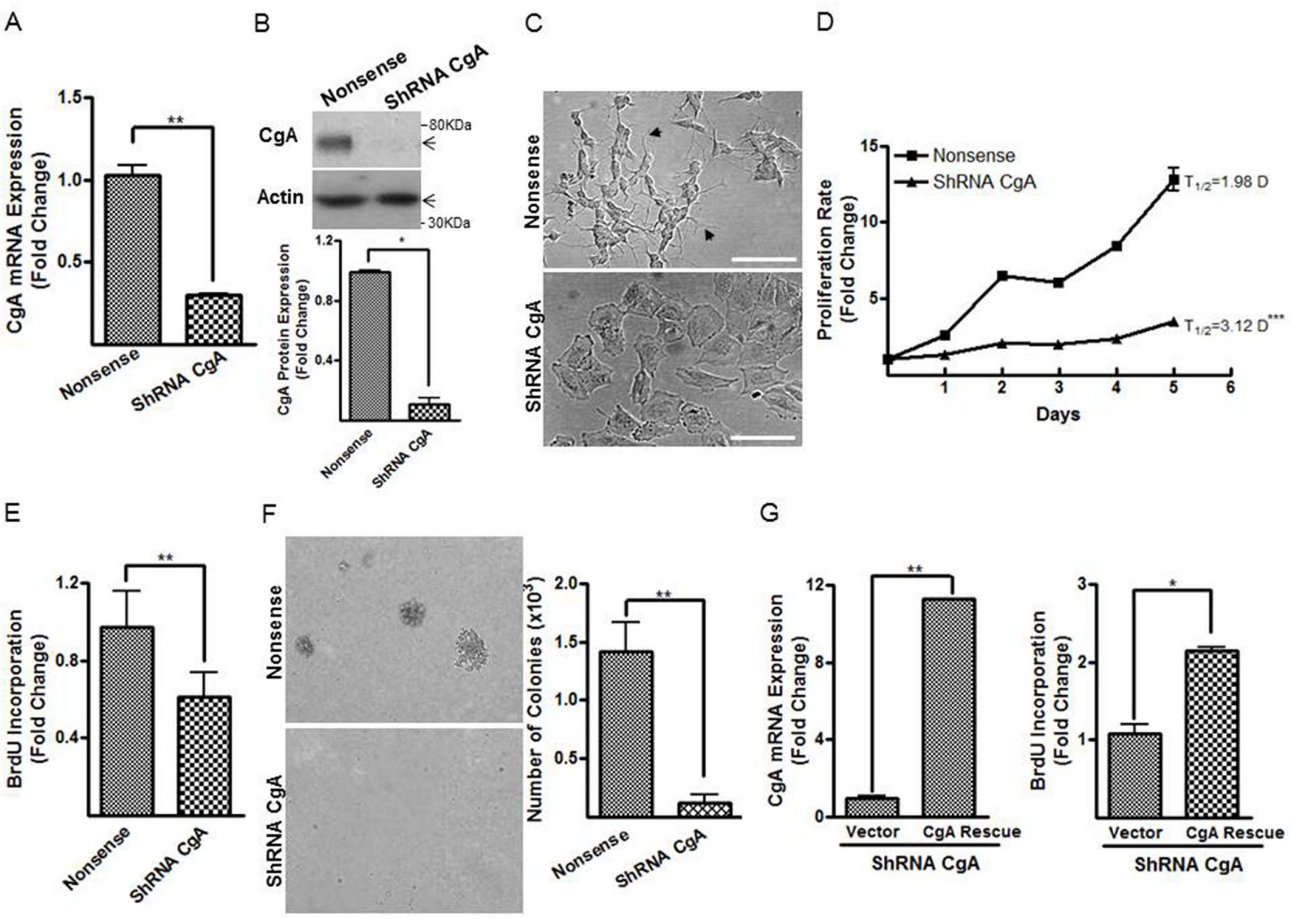
**CgA depletion inhibits cell proliferation and promotes cell differentiation in human neuroblastoma SH-SY5Y cells.** (A,B) CgA knockdown efficiency was confirmed by real-time PCR (A) and western blotting (B). The densitometric analyses of the protein bands versus the individual loading controls are shown under the blot. (C) Depiction of the morphological changes of the CgA knockdown neuroblastoma cells showing large, polygonal shaped cells compared to the smaller cells with short processing in controls. Scale bars: 100 µm. (D,E) Cell proliferation rates in the CgA knockdown and nonsense control neuroblastoma cells were measured by CellTiter-Glo^®^ luminescent cell viability assay (D) and BrdU incorporation (E). (F) Depiction of colonies in SH-SY5Y shRNA CgA and nonsense control cells in a soft agar assay to quantitate anchorage-independent tumor growth. (G) An shRNA-resistant CgA plasmid was transfected into SH-SY5Y shRNA CgA cells. Rescued CgA mRNA expression was confirmed by real-time PCR (left panel), and effect of CgA rescue in proliferation was evaluated by BrdU uptake assay (right panel). Normalization over nonsense control (A,B,E) or vector control (G) was used to calculate fold changes. Each bar indicates the mean±s.d. of triplicate tests. Data were analyzed by two-tailed unpaired *t*-test with Welch's correction, *P<0.05, **P<0.01; ***P<0.005.

To elucidate the biological function of CgA in modulation of neuroblastoma proliferation and differentiation, we used a short hairpin RNA (shRNA)-directed knockdown approach to deplete CgA expression in neuroblastoma SH-SY5Y cells *in vitro*. CgA knockdown efficiency was confirmed by real-time PCR [CgA mRNA expression (fold change), nonsense versus shRNA CgA, 1.0±0.1 versus 0.3±0.01, *P*<0.01, Fig. 1A] and western blotting [CgA protein expression (fold change), nonsense versus shRNA CgA 1.0±0.01 versus 0.1±0.03, *P*<0.05, Fig. 1B]. SH-SY5Y cells comprise three distinct morphologic phenotypes, among which N-type is the major type, followed by S- and I-type (Ross et al., 2003). We first observed morphological change of shRNA CgA cells compared to nonsense controls. The SH-SY5Y nonsense control cells displayed a typical N-type morphology predominantly characterized by an extensive network of neurite-like cytoplasmic processes (Fig. 1C, top panel). In contrast, SH-SY5Y shRNA CgA transfectants exhibited enlarged, firmly attached, polygonal shaped cells with occasional short processes (Fig. 1C, bottom panel). Concomitantly, a dramatic reduction in cell proliferation rates was observed in the shRNA CgA knockdown cells compared to nonsense control neuroblastoma cells as measured by the CellTiter-Glo^®^ luminescent cell viability assay (Fig. 1D) and BrdU incorporation assay (nonsense versus shRNA CgA, 1.0±0.1 versus 0.6±0.05, *P*<0.01, Fig. 1E). The calculated doubling time of CgA knockdown neuroblastoma cells was 1.5-fold longer than the nonsense control cells [T_1/2_ (days), nonsense versus shRNA CgA, 2.0 versus 3.1, *P*<0.005, Fig. 1D]. We also used Caspase-3 activation assay to determine the effect of CgA knockdown in cell survival, but no difference in cell death was observed (data not shown), indicating a pro-proliferative effect of CgA. In soft agar assays, fewer colonies formed in the SH-SY5Y shRNA CgA transfectants compared to nonsense control cells (number of colonies, nonsense versus shRNA CgA, 1416±254 versus 118±72, *P*<0.01, Fig. 1F), indicating that anchorage-independent growth was markedly impaired in the CgA knockdown cells. To further substantiate the shRNA-mediated CgA knockdown effect in neuroblastoma proliferation, we performed rescue experiment using an shRNA-resistant CgA plasmid which contained optimized CgA codon sequences to avoid recognition and degradation by the CgA shRNA [CgA mRNA expression (fold change), vector versus CgA Rescue, 1.0±0.1 versus 11.3±0.04, *P*<0.01, Fig. 1G, left panel]. Rescue re-expression of CgA in the shRNA CgA neuroblastoma cells resulted in increased BrdU incorporation (vector versus CgA rescue, 1.1±0.1 versus 2.1±0.05, *P*<0.05, Fig. 1G, right panel). These findings demonstrated that shRNA-directed CgA depletion reduced cell proliferation, inhibited anchorage-independent growth, and resulted in a striking morphology change in the human neuroblastoma SH-SY5Y cells *in vitro*.

### CgA knockdown alters phenotype of neuroblastoma cells

**Fig. 2.**
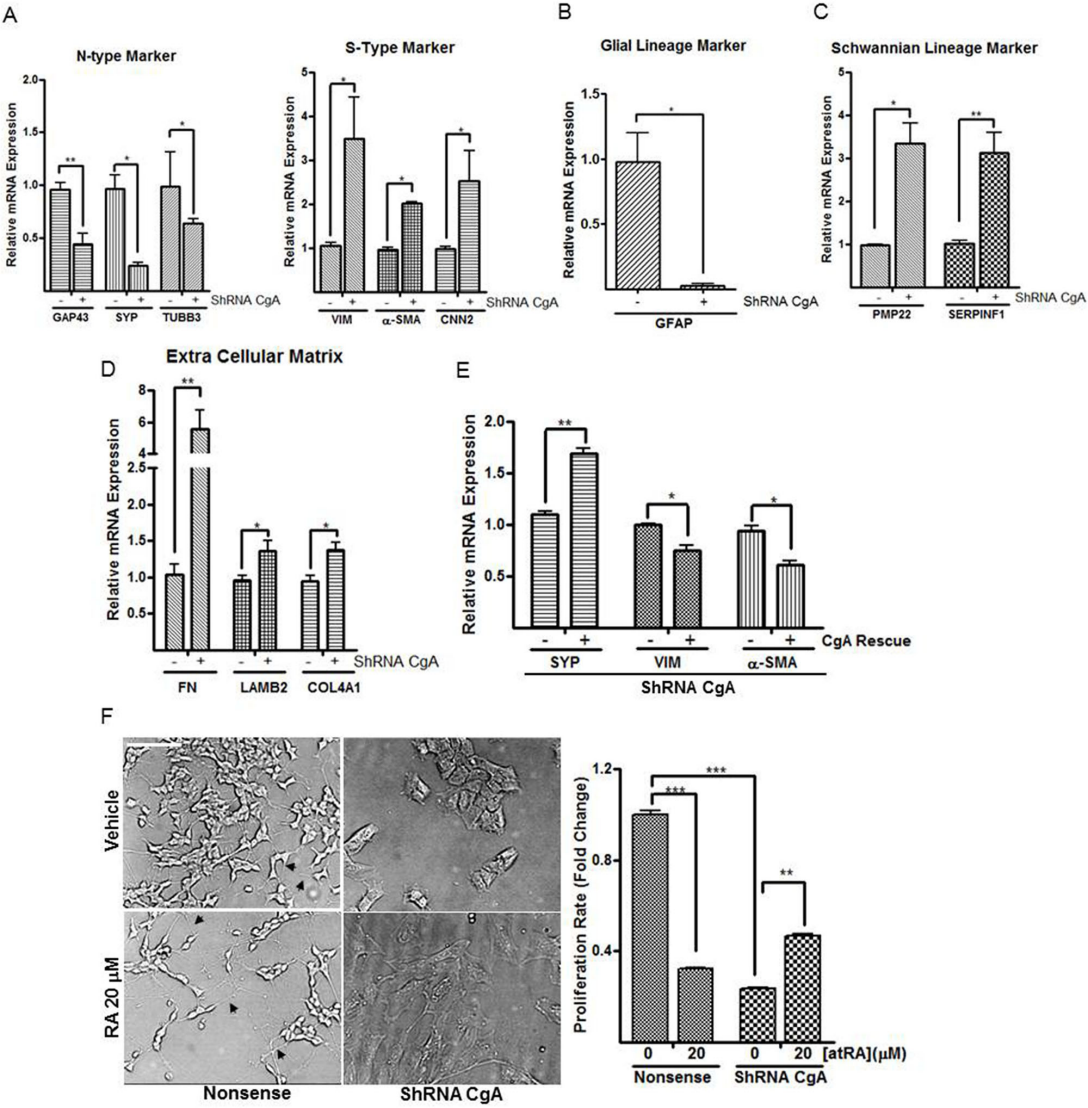
**ShRNA-directed CgA depletion promotes Schwann cell differentiation in human neuroblastoma SH-SY5Y cells.** (A) Quantitative PCR (qPCR) results depicting reduced N-type markers growth associated protein (GAP43), synaptophysin (SYP), and tubulin beta 3 (TUBB3) and increased S-type markers Vimentin (VIM), α-smooth muscle actin (α-SMA), and basic calponin (CNN2) mRNA levels in shRNA CgA knockdown cells compared to nonsense control neuroblastoma SH-SY5Y cells. (B–D) qPCR results depicting reduced expression of the glial cell marker (GFAP) (B), but increased expression of Schwannian cell lineage markers, peripheral myelin protein 22 (PMP22) (C) and serpin peptidase inhibitor (SERPINF1) (C), and Schwann cell related extracellular matrix genes, including fibronectin (FN), laminin beta 2 (LAMB2) and type IV collagen (COL4A1) (D) in shRNA CgA knockdown cells compared to nonsense control neuroblastoma SH-SY5Y cells. (E) CgA rescue experiment using an shRNA-resistant CgA plasmid to characterize phenotypic lineage marker changes (SYP for N-type, and VIM and α-SMA for S-type) by real-time PCR. Normalization over nonsense control (A–D) or vector control (E) was used to calculate fold changes. (F) All-trans retinoic acid (atRA)-treatment (20 µM) associated neurite outgrowth was observed in nonsense control neuroblastoma SH-SY5Y cells but not in the shRNA CgA cells treated with atRA. Scale bar: 100 µm, left panel. AtRA-induced cell growth arrest was abolished in shRNA CgA knockdown cells compared to nonsense control neuroblastoma cells (right panel). Proliferation rate fold change was relative luminescence signal to medium control of the nonsense control cells. Each bar indicates the mean±s.d. of triplicate tests. Data were analyzed by two-tailed unpaired *t*-test with Welch's correction, *P<0.05; **P<0.01; ***P<0.005.

Depending on local micro-environmental factors, neuronal crest precursor cells can give rise to progenies with different cell fates. These include neurons, glial cells, Schwann cells, adrenomedullary cells, melanocytes, chondrocytes, pericytes and smooth muscle cells of the vascular system (Abzhanov et al., 2003). To better understand the apparent phenotypic change we had observed in the CgA knockdown neuroblastoma cells, we next evaluated expression of several N-type cell lineage markers, including growth associated protein (GAP43), synaptophysin (SYP) and tubulin beta 3 (TUBB3), and several S-type lineage markers, including Vimentin (VIM), α-smooth muscle actin (α-SMA), and basic calponin (CNN2) (Sugimoto et al., 2000). Real-time PCR analysis demonstrated that all three N-type cell markers were reduced in the CgA knockdown SH-SY5Y cells (relative mRNA expression, nonsense versus shRNA CgA, GAP43 1.0±0.1 versus 0.4±0.1, *P*<0.01; SYP 1.0±0.1 versus 0.2±0.03, *P*<0.05; TUBB3 1.0±0.3 versus 0.6±0.04, *P*<0.05, Fig. 2A, left panel), whereas expression of S-type cell markers was increased in the CgA knockdown in comparison to nonsense control neuroblastoma cells (VIM 1.1±0.1 versus 3.5±1.0; α-SMA 1.0±0.1 versus 2.0±0.02; CNN2 1.0±0.1 versus 2.5±0.7, *P*<0.05, Fig. 2A, right panel), indicating that CgA knockdown promotes S-type cell commitment rather than an N-type cell fate. To further define which subset of S-type cells loss of CgA expression resulted in (Ciccarone et al., 1989), we then evaluated the glial cell and Schwannian cell lineage specific markers, glial fibrillary acidic protein (GFAP) (Abzhanov et al., 2003), peripheral myelin protein 22 (PMP22) (Magyar et al., 1996) and serpin peptidase inhibitor (SERPINF1) (Crawford et al., 2001). Expression of GFAP was reduced (nonsense versus shRNA CgA, 1.0±0.1 versus 0.02±0.01, *P*<0.05, Fig. 2B), whereas expression of PMP22 (1.0±0.02 versus 3.3±0.5, *P*<0.05, Fig. 2C) and SERPINF1 (1.0±0.1 versus 3.1±0.5, *P*<0.01, Fig. 2C) was increased in the shRNA CgA transfectants compared to nonsense control neuroblastoma cells. The pattern of alterations we observed in the cell lineage makers suggested that the SH-SY5Y shRNA CgA cells manifested a Schwannian cell type differentiation. We further demonstrated that three extracellular matrix (ECM) genes synthesized by Schwannian cells, namely fibronectin (FN), laminin beta 2 (LAMB2) and type IV collagen (COL4A1) (Tsokos et al., 1985), were increased in the SH-SY5Y shRNA CgA cells compared to nonsense control neuroblastoma cells (relative mRNA expression, nonsense versus shRNA CgA, FN 1.0±0.1 versus 5.6±1.2, *P*<0.01; LAMB2 1.0±0.1 versus 1.4±0.1, *P*<0.05; COL4A1 0.9±0.1 versus 1.4±0.1, *P*<0.05, Fig. 2D). As further evidence of our observed phenotypic change, following CgA knockdown in neuroblastoma cells, we also performed rescue experiment to restore CgA expression. We demonstrated that expressing shRNA-resistant CgA in SH-SY5Y CgA knockdown cells increased N-type marker SYP (1.1±0.03 versus 1.7±0.05, *P*<0.01, Fig. 2E) and decreased S-type markers VIM (1.0±0.01 versus 0.75±0.05, *P*<0.05, Fig. 2E) and α-SMA (0.9±0.04 versus 0.6±0.03, *P*<0.05, Fig. 2E) expressions. Prior studies have documented the actions of all-trans retinoic acid (atRA) to induce morphological differentiation of neuroblastoma cells towards a neuronal lineage (Gaetano et al., 1992). We observed that whereas atRA treatment caused obvious neurite outgrowth in nonsense control neuroblastoma cells as previously reported, treatment of the shRNA CgA transfectants with atRA (20 µM) did not change the S-type morphology (Fig. 2F, left panel). Furthermore, although atRA treatment inhibited *in vitro* neuroblastoma proliferation in the nonsense control neuroblastoma cells (nonsense, vehicle versus atRA, 1.0±0.02 versus 0.32±0.001, *P*<0.005, Fig. 2F, right panel), atRA treatment did not inhibit but increased proliferation in the SH-SY5Y shRNA CgA cells (shRNA CgA, vehicle versus atRA, 0.24±0.001 versus 0.45±0.01, *P*<0.01, Fig. 2F, right panel), further emphasizing the difference in cell phenotype induced by CgA knockdown.

### Confirmation of the role of CgA in cell proliferation and differentiation in multiple neuroblastoma cell lines

**Fig. 3.**
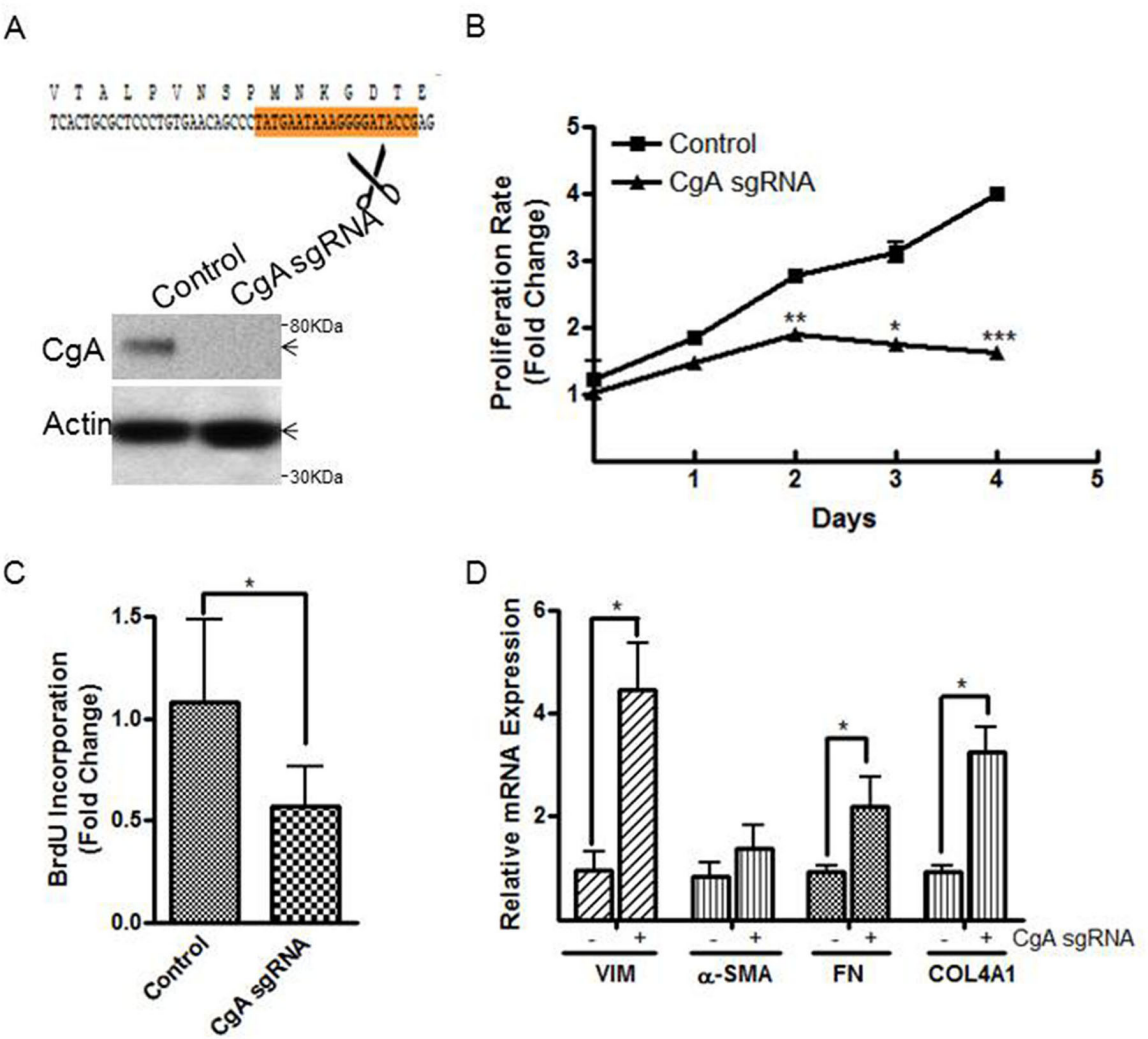
**CRISPR-Cas9-mediated knockout confirmed the role of CgA in cell proliferation and differentiation.** (A) Depiction of the targeting site on CgA Exon 2 chosen for CRISPR/Cas9-directed CgA knockout (top panel), which was confirmed by western blotting (bottom panel). (B,C) CgA sgRNA transfectants exhibited lower proliferation rates compared to control neuroblastoma SH-SY5Y cells measured by CellTiter-Glo^®^ luminescent cell viability assay (B) and BrdU incorporation (C). (D) Quantitative PCR demonstrated that the CgA sgRNA knockout neuroblastoma cells exhibited increased S-type markers (VIM and α-SMA), ECM markers (FN and COL4A1) compared to control neuroblastoma transfectants. Normalization over control cells (B–D) was used to calculate fold changes. Each bar indicates the mean±s.d. of triplicate tests. Data were analyzed by two-tailed unpaired *t*-test with Welch's correction, *P<0.05; **P<0.01; ***P<0.005.

**Fig. 4.**
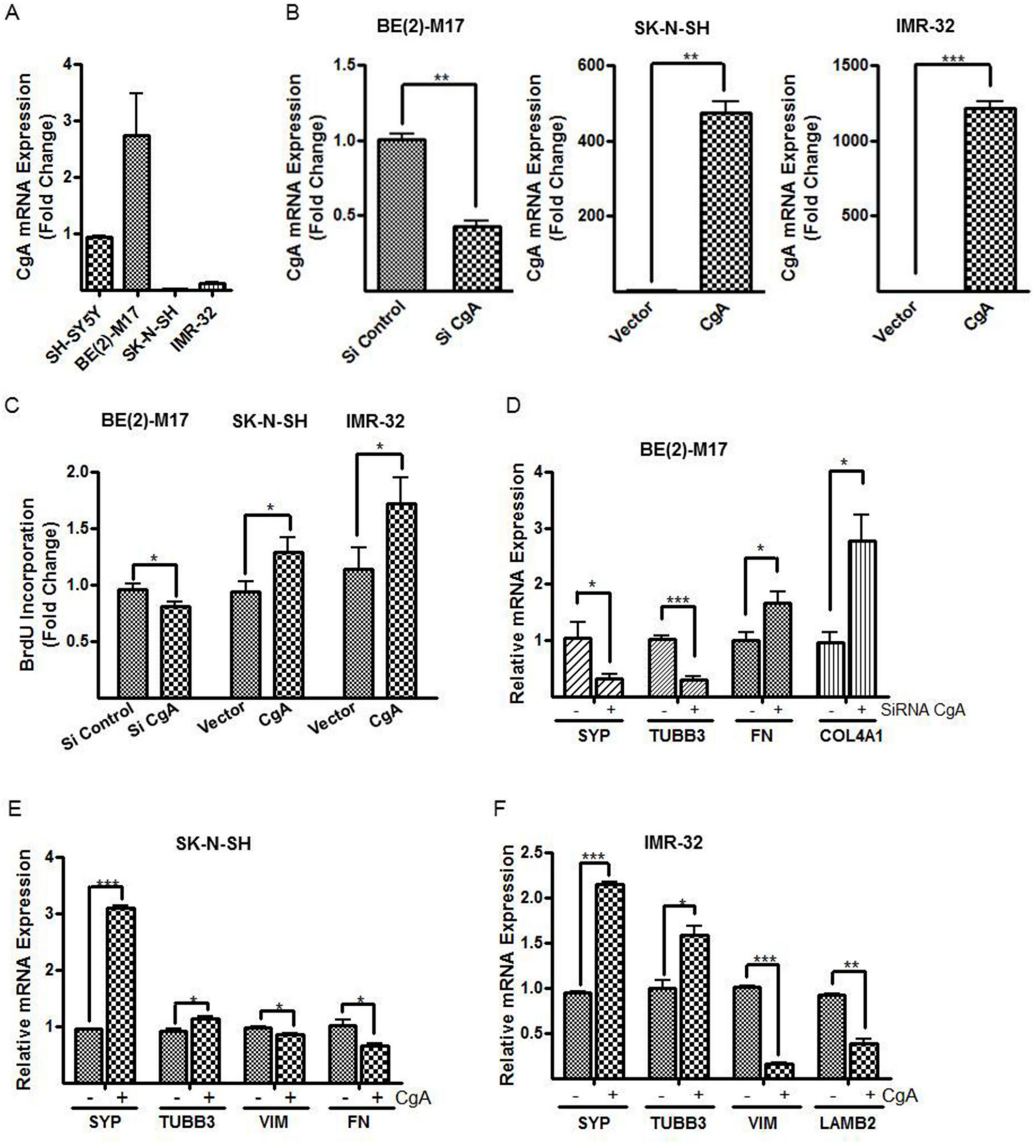
**Effects of CgA in cell proliferation and phenotypic changes in additional neuroblastoma cell lines.** (A) CgA mRNA expression was evaluated in a series of neuroblastoma cell lines with (BE(2)-M17 and IMR-32) and without (SH-SY5Y and SK-N-SH) N-Myc amplification. Relative CgA mRNA expression was calculated using the 2^−ΔΔ*CT*^ method normalized to that in SH-SY5Y cells. (B) SiRNA CgA and SiRNA control were transfected into BE(2)-M17 and hCgA-pCMV6-Entry plasmid and empty vector were transfected in SK-N-SH and IMR-32 cells for knockdown and overexpression experiments respectively. 24 h later, the cells were collected to analyze CgA expression by real-time PCR. (C) The effects of CgA knockdown and overexpression in proliferation rates in BE(2)-M17, SK-N-SH and IMR-32 cells were measured by BrdU incorporation assay. (D–F) Cell linage specific markers were examined following CgA knockdown in BE(2)-M17 cells (D), CgA overexpression in SK-N-SH (E) and IMR-32 (F) cells by real-time PCR. Normalization over siRNA control or vector control was used to calculate fold changes (B–F). Each bar indicates the mean±s.d. of triplicate tests. Data were analyzed by two-tailed unpaired *t*-test with Welch's correction, *P<0.05; **P<0.01; ***P<0.005.

To further support our findings on the actions of CgA to alter neuroblastoma phenotype and proliferation rates *in vitro*, we also used CRISPR-Cas9 to completely deplete CgA expression in the SH-SY5Y cells. We used a 20-bp region in CgA Exon 2 as a single guiding RNA (sgRNA) to direct Cas9-mediated insertions or deletions (indels) to knockout CgA in the SH-SY5Y cells, which was confirmed by western blotting (Fig. 3A). Complete CgA loss in SH-SY5Y cells led to marked inhibition of *in vitro* proliferation measured by CellTiter-Glo^®^ luminescent cell viability assay (Fig. 3B) and BrdU incorporation assay (control versus CgA sgRNA, 1.1±0.2 versus 0.57±0.08, *P*<0.05, Fig. 3C). We also observed the same altered phenotype in the CgA knockout cells with differentiation toward an S-type as evidenced by increased VIM, FN and COL4A1 mRNA expression detected by real-time PCR in the CgA knockout cells compared to control cells (relative mRNA expression, control versus CgA sgRNA, VIM 1.0±0.3 versus 4.5±0.9; FN 0.9±0.1 versus 2.2±0.6; COL4A 0.9±0.1 versus 3.2±0.5, *P*<0.05, Fig. 3D). These knockout studies corroborated our prior findings using shRNA and demonstrated that reducing CgA expression in neuroblastoma SH-SY5Y cells inhibits *in vitro* cell proliferation and promotes cell differentiation toward a Schwannian cell phenotype. To evaluate the role of CgA more broadly in neuroblastoma, we compared endogenous CgA expression in three additional cell lines with (BE(2)-M17 and IMR-32) or without (SK-N-SH) N-Myc amplification. We found that BE(2)-M17 together with SH-SY5Y cells exhibited significantly higher CgA expression than SK-N-SH and IMR-32 cells [CgA mRNA expression (fold change), SH-SY5Y 0.9±0.05, BE(2)-M17 2.7±1.3, SK-N-SH 0.005±0.0006, IMR-32 0.1±0.01, Fig. 4A]. We used SiRNA to knockdown CgA in BE(2)-M17 (CgA mRNA fold change, SiRNA control versus SiRNA CgA, 1.0±0.03 versus 0.4±0.04, *P*<0.01, Fig. 4B, left panel) and overexpressed CgA in SK-N-SH (vector versus CgA, 1.0±0.1 versus 473±51, *P*<0.01, Fig. 4B, middle panel) and IMR-32 cells (1.1±0.2 versus 1217±74, *P*<0.005, Fig. 4B, right panel) respectively by transfecting a hCgA-pCMV6-Entry plasmid. The CgA knockdown in BE(2)-M17 cells exhibited 20% reduced proliferation [BrdU incorporation (fold change), SiRNA control versus SiRNA CgA, 1.0±0.03 versus 0.8±0.02, *P*<0.05, Fig. 4C], while CgA overexpression increased proliferation by 40% in SK-N-SH (vector versus CgA, 0.9±0.04 versus 1.3±0.07, *P*<0.05, Fig. 4C) and IMR-32 cells (1.1±0.1 versus 1.7±0.2, *P*<0.05, Fig. 4C) respectively. CgA knockdown in BE(2)-M17 cells also led to reduced expression of the N-type cell markers (relative mRNA expression, SiRNA control versus SiRNA CgA, SYP 1.0±0.3 versus 0.3±0.07, *P*<0.05; TUBB3 1.0±0.1 versus 0.3±0.06, *P*<0.005, Fig. 4D), whereas the Schwannian associated ECM specific molecules were increased (FN 1.0±0.1 versus 1.7±0.2; COL4A1 1.0±0.2 versus 2.8±0.5, *P*<0.05, Fig. 4D). In contrast, CgA overexpression resulted in increased expression of N-type cell markers in SK-N-SH (relative mRNA expression, vector versus CgA, SYP 1.0±0.02 versus 3.1±0.03, *P*<0.005; TUBB3 0.9±0.04 versus 1.1±0.04, *P*<0.05, Fig. 4E) and IMR-32 cells (SYP 0.95±0.02 versus 2.1±0.03, *P*<0.005; TUBB3 1.0±0.08 versus 1.6±0.1, *P*<0.05, Fig. 4F), while S-type marker VIM was reduced by CgA overexpression in SK-N-SH (vector versus CgA, 0.98±0.02 versus 0.86±0.02, *P*<0.05, Fig. 4E) and IMR-32 cells (1.0±0.01 versus 0.16±0.01, *P*<0.005, Fig. 4F). CgA overexpression also reduced ECM marker in SK-N-SH (relative FN mRNA expression, 1.0±0.1 versus 0.65±0.05, *P*<0.05, Fig. 4E) and IMR-32 cells (relative LAMB2 mRNA expression, 0.9±0.01 versus 0.4±0.05, *P*<0.01, Fig. 4F). Collectively, these results supported our initial findings in SH-SY5Y cells and confirmed the effects of CgA in cell proliferation and differentiation in neuroblastoma.

### CgA knockdown suppresses IGFR, AKT and MAPK activity, and IGF2 addition rescues the proliferation defects of the knockdown cells

**Fig. 5.**
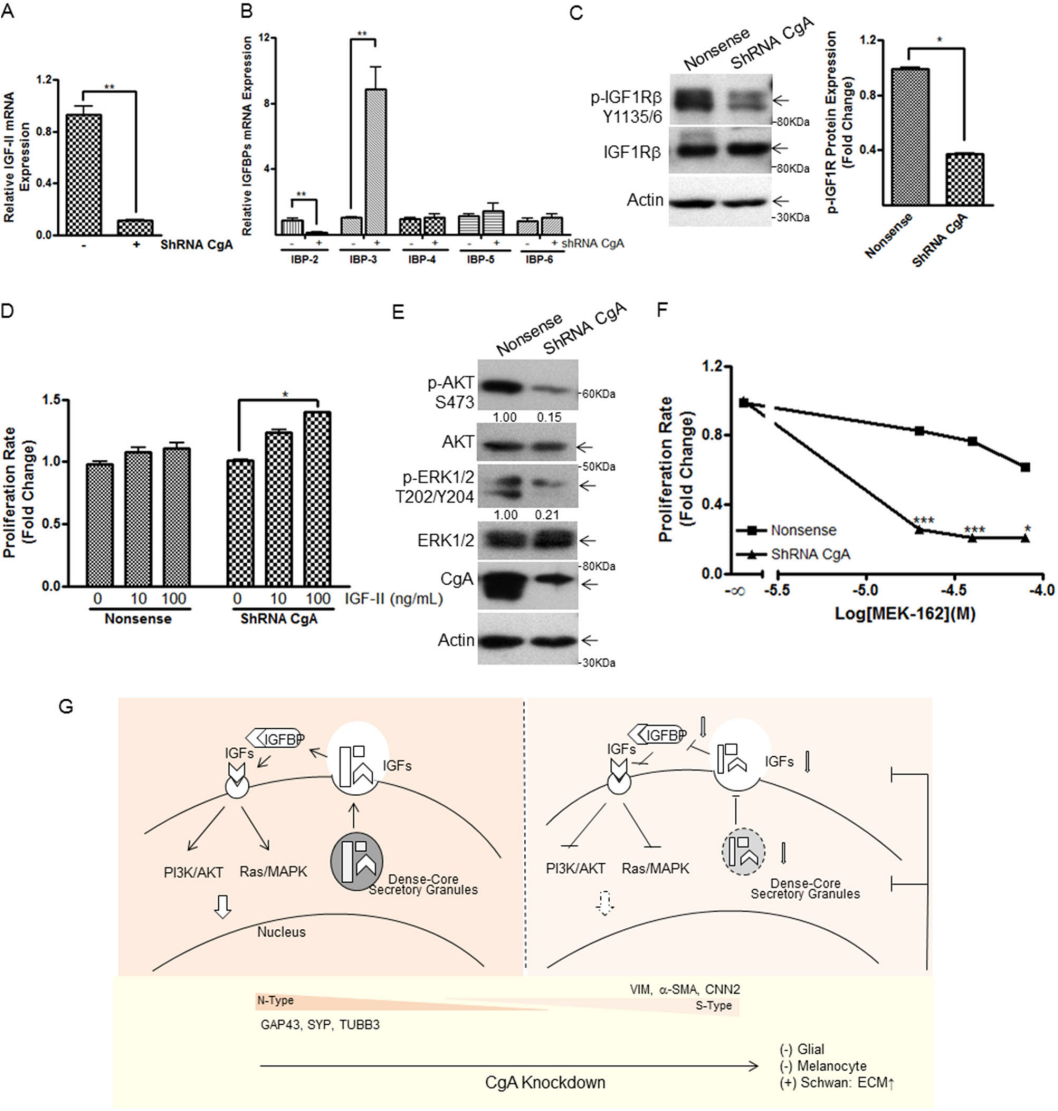
**CgA knockdown results in impairment in IGF signaling.** (A,B) Quantitative PCR depicting changes in IGF-II (A) and IFGBP-2, -3, -4, -5 and -6 expression (B) in shRNA CgA transfectants compared to nonsense control neuroblastoma SH-SY5Y cells. (C) Detection of phosphorylated IGF1R in shRNA CgA and nonsense control cells. The densitometric analyses of the protein bands versus the individual loading controls were shown in the right panel. (D) Comparison of the pro-proliferative effect of IGF-II in shRNA CgA and nonsense control cells by CellTiter-Glo^®^ luminescent cell viability assay. (E) Western blot depiction of reduced AKT/ERK pathway activation in shRNA CgA transfectants compared to nonsense control neuroblastoma cells. The densitometric analyses of the protein bands versus the individual loading controls are shown under the blot. (F) SH-SY5Y shRNA CgA transfectants were more responsive to the growth inhibitory action of ERK inhibitor compared to nonsense control neuroblastoma cells. (G) Schematic summary of our proposed actions of CgA depletion in neuroblastoma cells *in vitro* to promote a Schwannian phenotype via the reduced IGF signaling and PI3K/AKT/Ras/MAPK pathways. Normalization over nonsense control (A,B) or medium control (D,F) was used to calculate fold changes. Each bar indicates the mean±s.d. of triplicate tests. Data were analyzed by two-tailed unpaired *t*-test with Welch's correction, **P<0.01; ***P<0.005.

As noted above, CgA plays a crucial role in the biogenesis of secretory granules, and is involved in sorting and processing a wide spectrum of peptide hormones, amines and ions that maintain physiological homeostasis by both endocrine- and auto/paracrine-dependent actions (Bartolomucci et al., 2011). Insulin-like growth factors (IGFs) are intricately involved in regulation of tumor growth and differentiation, and previous studies have highlighted the role of IGF-II in neuroblastoma (Dake et al., 2004; Grellier et al., 2002). IGF binding protein-2 (IGFBP-2) expression frequently positively correlates with IGF-II levels and cell proliferation (El-Badry et al., 1991; Grellier et al., 2002). Secreted IGFs are non-covalently bound to IGFBP-1 to -6 with high affinity during transportation, and IGFBPs function as a reservoir to buffer IGF bioavailability, where IGFBP-3 is the predominant circulating IGF carrier (Tanno et al., 2004; Yu and Rohan, 2000). IGFBP-3-mediated actions in cancer vary in a disease-specific manner and some studies suggest that intracellular IGFBP-3 functions may be independent of its IGF binding ability (Baxter, 2014). We observed that knockdown of CgA in neuroblastoma SH-SY5Y cells resulted in a reduction in both IGF-II and IGFBP-2 mRNA expression (relative mRNA expression, nonsense versus shRNA CgA, IGF-II 0.9±0.1 versus 0.1±0.01, *P*<0.01, Fig. 5A; IGFBP-2 0.9±0.2 versus 0.1±0.1, *P*<0.01, Fig. 5B). CgA knockdown also led to increased IGFBP-3 expression (nonsense versus shRNA CgA, 1.0±0.1 versus 8.9±1.4, *P*<0.01, Fig. 5B). In contrast CgA knockdown did not alter expression of IGFBP-4, 5 or 6 (Fig. 5B), and expression of both IGF-1 and IGFBP-1 were under the detection limit of the assay in both the control and shRNA CgA SH-SY5Y cells (data not shown). IGF-II actions are transduced by cognate transmembrane receptor tyrosine kinases that include the tetrameric type I insulin-like growth factor receptor (IGF1R), the insulin receptor (INSR), and the monomeric IGF2R which lacks intracellular tyrosine kinase domain (Ryan and Goss, 2008). We next quantified phosphorylated IGF1R by western blot. As shown in Fig. 5C, lower phospho-IGF1Rβ (Y1135/1136) expression was seen in shRNA CgA cells compared to nonsense control cells, indicating attenuation of IGF pathway activation following CgA depletion. To further support a role for altered autocrine IGF-II proliferative actions following CgA depletion, we performed a ‘rescue’ experiment by treating both shRNA CgA and nonsense control SH-SY5Y neuroblastoma cells with exogenous IGF-II. As depicted in Fig. 5D, exogenous IGF-II treatment (100 ng/ml) resulted in increased proliferation in the ShRNA CgA cells compared to control cells (IGF-II versus medium control, nonsense 1.1±0.05 versus 1.0±0.01, *P*>0.05; shRNA CgA 1.4±0.003 versus 1.0±0.01, *P*<0.05, Fig. 5D).

As IGFs signal through the PI3K/AKT/MAPK pathways, we next examined phosphorylated AKT and ERK1/2 expression in our CgA knockdown cells. As depicted in Fig. 5E, CgA knockdown led to reduced phosphorylated AKT and phosphorylated ERK1/2 protein expression compared to that observed in control cells. To extend these findings, we next used ERK inhibitor (MEK-162) and demonstrate the treatment caused greater inhibition of cell proliferation in shRNA CgA compared to nonsense control cells (nonsense versus shRNA CgA, MEK-162 20µM, 0.7±0.01 versus 0.3±0.003, *P*<0.005; MEK-162 40 µM, 0.5±0.01 versus 0.2±0.001, *P*<0.005; MEK-162 80 µM, 0.3±0.01 versus 0.2±0.001, *P*<0.05 Fig. 5F). An illustration of the *in vitro* effects we have observed following neuroblastoma CgA depletion is described in Fig. 5G with reduced expression of IGF-II and IGFBP-2, combined alteration of which may contribute to reduced growth factor signaling as evidenced by reduced p-IGF1R signaling and increased responsivity to pharmacological inhibitor.

### Flank xenografts of neuroblastoma cells lacking CgA show a shift towards an S-phenotype

**Fig. 6.**
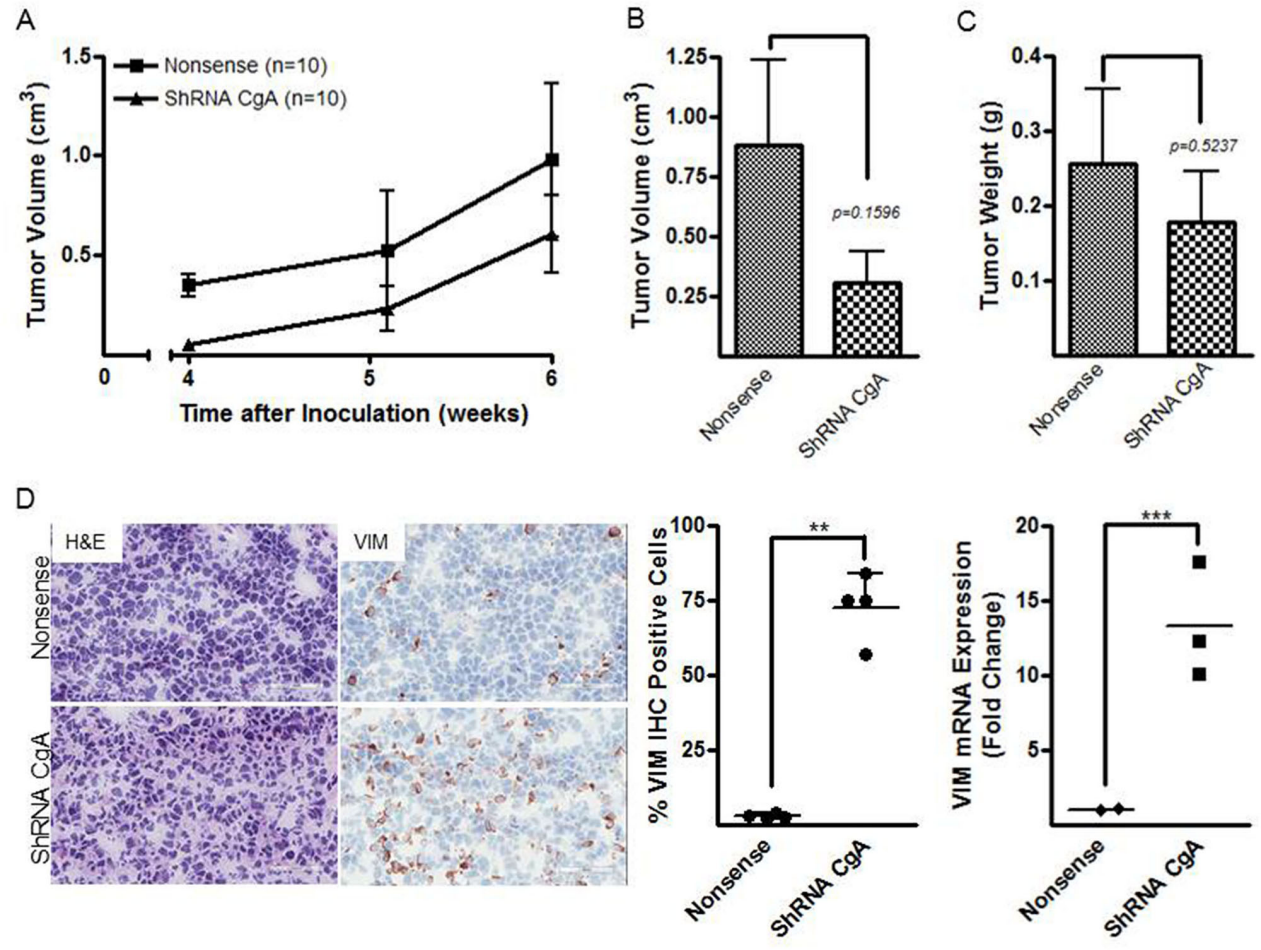
**Flank xenografts of neuroblastoma cells lacking CgA show a shift towards an S-phenotype.** (A) Comparison of tumor development time in CgA knockdown cells (*n*=10) and nonsense control neuroblastoma cells (*n*=10) in an *in vivo* xenograft model of neuroblastoma. Trend towards a reduction in tumor volumes (B) and weights (C) in the animals bearing CgA knockdown cells compared to nonsense control carrying animals. Note that these results did not attain statistical significance. (D) Representative images of tumor H&E and Vimentin IHC staining (*n*=4 for each group, left panel, scale bars: 50 mm), percentages of VIM immunoreactive cells (middle panel), and VIM mRNA expression (*n*=2 for nonsense group, and *n*=3 for shRNA CgA group, right panel) in two groups. Each bar indicates the mean±standard deviation of triplicate tests. Data were analyzed by two-tailed unpaired *t*-test with Welch's correction, **P<0.01; ***P<0.005.

We next tested effects of CgA depletion in neuroblastoma tumor growth *in vivo*. Human neuroblastoma SH-SY5Y cells stably transfected with shRNA CgA or nonsense control were subcutaneously inoculated into five-week-old male athymic nude mice at the density of 1×10^6^ cells/animal. Mice were examined for tumor presence twice a week, and tumor volume was measured following palpable tumor development 4 weeks after tumor injection. The mice inoculated with SH-SY5Y shRNA CgA developed tumors later than the mice inoculated with nonsense control neuroblastoma cells (Fig. 6A). By 6 weeks after tumor injection, the animals were euthanized as tumors in the control group became debilitating. Tumor volume in the SH-SY5Y shRNA CgA group was reduced compared to that of nonsense control group [tumor volume (cm^3^), nonsense versus shRNA CgA, 0.9±0.4 versus 0.3±0.1, *P*=0.1596, Fig. 6B]. The average tumor weight in shRNA CgA group was also reduced [tumor weight (g), nonsense versus shRNA CgA, 0.3±0.1 versus 0.2±0.1, *P*=0.5237, Fig. 6C], although these differences did not attain statistical significance. Immunohistochemical analysis for the S-type marker VIM in frozen tumor tissues demonstrated VIM was expressed in 72.7±5.7% cells from SH-SY5Y shRNA CgA bearing animals (*n*=4) compared to 2.6±0.5% in SH-SY5Y nonsense control tumors (*n*=4, *P*<0.01, Fig. 6D), and VIM mRNA expression was also elevated in tumors from shRNA CgA group (*n*=3) compared to those from nonsense control (*n*=2, nonsense versus shRNA CgA 1.0±0.1 versus 13.3±1.1, *P*<0.005, Fig. 6D). These results corroborated our *in vitro* findings that CgA loss results in a shift towards an S-phenotype.

## DISCUSSION

Neuroblastoma is a commonly encountered solid tumor in early childhood with a prevalence of ∼1 case per 8000 live births, and an annual incidence of ∼10 cases/million children aged <15 years (Ratner et al., 2016). Approximately half of these neuroblastoma tumors are classified as high-risk, and despite intensive multimodal therapy, including surgery, radiation and chemotherapy, the overall survival rate of this group of patients is <40% (Ferrari-Toninelli et al., 2010; Sun et al., 2013). Thus innovative therapeutic approaches are urgently needed to successfully treat this disease (Karmakar et al., 2011). Neuroblastoma arises from tissues derived from the neural crest and it is proposed that cells that become arrested along this migratory differentiation pathway develop into neuroblastomas (Thiele, 1990). Given the apparent multipotent neuroplasticity of the disease, it has been hypothesized that therapy which promotes differentiation and commitment toward specific cell lineages could lead to tumor shrinkage and/or relief of symptoms (Ratner et al., 2016). 13-Cis-retinoic acid, which induces a neuronal cell linage differentiation, is the most widely used agent in this regard, but after an initial response, chemo-resistance and tumor relapse is often seen (Lasorella et al., 1995). Better understanding of the mechanisms involved in differentiating those tumors could ultimately foster novel treatment approaches for neuroblastoma.

CgA is a tissue specific protein restricted to the diffuse neuroendocrine system, and widely expressed in neuroblastomas. Prior studies have demonstrated reduced CgA transcription following atRA and 12-O-tetradecanoylphorbol-13-acetate (TPA)-induced neuroblastoma neuronal differentiation, concomitant with growth arrest (Gaetano et al., 1995). Conversely, glucocorticoid-treatment *in vitro* increased CgA expression and promoted chromaffin cell differentiation accompanied by increased N-Myc expression, a well characterized indicator of a poor prognosis (Ross et al., 2002; Rozansky et al., 1994). Underpinning the clinical relevance of this finding, a primary neuroblastoma located in or near the adrenal gland is often a higher grade tumor with a two-year survival rate of less than 20% (Ross et al., 2002). N-Myc amplification is prevalent in this group (Ross et al., 2002), and it has been proposed that the high regional steroid concentrations from the adjacent adrenal cortex inhibit sympathoblast neuronal differentiation and promote chromaffin maturation, resulting in a more aggressive disease phenotype (Gestblom et al., 1997). Furthermore, the glucocorticoid receptor antagonist RU-486 has been observed to decrease neuroblastoma cell proliferation (Maggi et al., 1998; Sengupta et al., 2000). Our studies herein show that CgA depletion using knockdown and knockout approaches resulted in reduced cell proliferation and promoted differentiation toward a Schwannian cell phenotype. Schwann cells are considered part of the stromal element in neuroblastoma and stroma-rich tumors have a more favorable prognosis compared with stroma-poor tumors (Ross et al., 2003). It has also been shown that the interplay between tumor and Schwann cells dynamically shapes neuroblastoma differentiation and disease outcome (Ross et al., 2003). For example, Schwann cell proliferation is promoted by the growing axons from differentiating neuroblastoma cells through direct contact and production of soluble chemotactic or mitogenic factors (Ambros et al., 2001). In parallel, Schwann cells secrete soluble factors and extracellular matrix that can promote tumor differentiation and inhibition of growth and angiogenesis (Ambros and Ambros, 1995). Our findings demonstrate that CgA depletion in neuroblastoma cells resulted in a cell morphological shift associated with increased expression of Schwann and ECM specific molecules (PMP22, SERPINF1, FN, LAMB2 and COL4A1), and suppression of features associated with the chromaffin phenotype (reduced IGF-II).

The MAPK/ERK signaling cascade is under the control of multiple extracellular ligands, mitogens and growth factors, including the insulin like growth factor family (IGFs). In some types of mesenchymal cells, such as bone marrow mesenchymal stem cells, constitutive activation of the MAPK/ERK cascade blocks differentiation into a smooth muscular lineage (Tamama et al., 2008). Confirming prior studies in other tumor types, we observed reduced AKT/ERK activation following CgA knockdown in neuroblastoma cells (Gong et al., 2007; Khan et al., 2012; Yu et al., 2003). Additionally, we demonstrated reduced IGF-II expression in CgA depleted cells and exogenous IGF-II treatment caused greater pro-proliferation effect in ShRNA CgA cells in comparison to control cells, which leads to our proposal that loss of CgA impairs IGF-II-mediated autocrine and paracrine proliferation signaling. This in turn leads to increased sensitivity to the anti-proliferative effect of chemical inhibitor that we observed in the shRNA CgA knockdown neuroblastoma cells.

In summary, we demonstrated that depletion of CgA in neuroblastoma inhibits cell proliferation and leads to Schwannian differentiation *in vitro*. CgA depletion also led to impaired IGF autocrine signaling with reduced AKT/ERK pathway activation, which may underlie the molecular basis for our observed findings. Although we did observe reduced tumor growth *in vivo* in the shRNA CgA knockdown neuroblastoma cells, this did not attain statistical significance. This may have occurred for several reasons. Firstly, the number of animals was small and our study may not have been sufficiently statistically powered. Secondly, given that CgA is an ubiquitous protein and the growth factors such as IGFs are secreted by endothelial and other cells, the tumor *in vivo* microenvironment may have been able to compensate for the CgA-related loss. However, given the striking alterations we observed of CgA depletion *in vitro* with a marked phenotypic shift, we still feel that our findings are significant and could enable novel therapeutic approaches in neuroblastoma differentiation therapy.

## MATERIALS AND METHODS

### Cell culture and reagents

Human neuroblastoma SH-SY5Y (purchased from ATCC, CRL­2266), BE(2)-M17 (from ATCC, CRL-2267), SK-N-SH and IMR-32 cells (kindly provided by Dr M. Sue O'Dorisio, Department of Pediatrics, University of Iowa) (Sue O'Dorisio et al., 1992) were cultured as monolayers at 37°C, 5% CO_2_ using 1:1 mixture of ATCC-formulated Eagle's Minimum Essential Medium (30-2003) and F12 Medium containing 10% fetal bovine serum (FBS), and penicillin/streptomycin. They were recently authenticated and tested for mycoplasma contamination. The cultures were detached with trypsin and transferred to new 75-cm^2^ culture flasks (Thermo Fisher Scientific) once a week. DMEM, FBS and antibiotics were purchased from Life Technologies, Inc. For *in vitro* cell culture studies, all trans-retinoic acid (atRA) and MEK-162 were solved in DMSO, and IGF-II (Thermo Fisher Scientific) was solved in DMEM/F12 at concentrations of 10 mM and 0.1 mg/ml respectively and stored in −80C freezer in aliquots, and later diluted as described in experiments.

### Plasmid constructs and transfection

ShRNA CgA (V2LHS_112999) and non-silencing control (RHS4346), CgA siRNA (L-011240-00-0005) and non-targeting Pool (D-001810-10-05) were purchased from GE Healthcare Dharmacon, Inc. (Lafayette, CO). sgRNA/Cas9 all-in-one expression clone targeting CHGA (NM_001301690.1) and scrambled sgRNA control were obtained from GeneCopoeia, Inc. (Rockville, MD). Human CgA overexpression plasmid hCgA-pCMV6-Entry (RC200492) was purchased from OriGene Technologies, Inc. (Rockville, MD). ShRNA-resistant human CgA overexpression plasmid containing codon optimized sequences (mutated from 63-CCT GTG AAC AGC CCT-75 to 63-CCC GTC AAT AGT CCG-75) to prevent destruction by the shRNA was synthesized by Genewiz (South Plainfield, NJ). All constructs were verified by sequencing. Lipofectamine 2000 (Invitrogen) was used for transfection according to manufacture instruction. The stable shRNA CgA and nonsense control neuroblastoma cells were established by puromycin selection (0.5 µg/ml) for 3 weeks. The CgA sgRNA knockout cells were established by cloning after two-day selection with G418 (2000 µg/ml). SiRNA CgA and SiRNA control were transfected into BE(2)-M17 cells for knockdown experiment. hCgA-pCMV6-Entry plasmid was transfected into SK-N-SH and IMR-32 cells for the overexpression experiment, and ShRNA-resistant human CgA plasmid was transfected in SH-SY5Y shRNA CgA cells for the rescue experiment. 24–72 h post transfection, the cells were harvested for cell proliferation assay and gene expression analysis by real-time PCR.

### CellTiter-Glo^®^ proliferation assay

Cells were suspended in 100 μl DMEM supplemented with 10% FBS, and plated in 96-well plates (2×10^4^ viable cells/well) and cultured overnight. Cell viability was determined using CellTiter-Glo^®^ Luminescent Cell Viability Assay kit (Promega) with a luminometer (Wallac 1420 Victor 2 multipliable counter system). Results are presented as proliferation rate fold change (relative luminescence signal to nonsense control or medium control as indicated) and all experiments were repeated at least three times and presented as mean±s.d.

### BrdU cell proliferation assay

Cell proliferation was quantified in 96-well plate (2×10^4^ cells/well) using a colorimetric BrdU Cell Proliferation ELISA Kit (Abcam, ab126556) according to manufacture instruction. Results are presented as BrdU incorporation fold change (relative absorbance at 450 nm to nonsense control as indicated) and all experiments were repeated at least three times and presented as mean±s.d.

### Anchorage-independent growth assay

Anchorage-independent growth (soft agar assay) was performed as described in our previous studies (Zhang et al., 2009). Briefly, 1×10^5^ cells suspended in 0.33% soft agar were seeded over a bottom layer of 0.5% agar in 10% FBS DMEM in each well of six-well plates. The plates were incubated in 5% CO_2_ incubator at 37°C for 3 weeks. Colonies were inspected under a microscope and only colonies with ≥32 cells were counted and all experiments were repeated at least three times and presented as mean±s.d.

### Real-time PCR

Total RNA was extracted with RNeasy kit (Qiagen). RNA quantification and integrity were assessed by measurement of absorbance at 260 and 280 nm. Total RNA was reverse transcribed into first-strand cDNA using a cDNA synthesis kit (Invitrogen). Quantitative PCR reactions were carried out using CFX Real-time PCR Detection System (Bio-Rad). Primer sequences (Invitrogen/Life Technologies) were as follows: human *CgA* forward primer, 5′-AAG AGA GGA TTC CAA GGA GGC-3′; human *CgA* reverse primer, 5′-TGA TTG TTC CCC TCA GCC TTG-3′; human *GAP43* forward primer, 5′-GAG CAG CCA AGC TGA AGA GAA C-3′; human *GAP43* reverse primer, 5′-GCC ATT TCT TAG AGT TCA GGC ATG-3′; human *SYP* forward primer, 5′-TCG GCT TTG TGA AGG TGC TGC A-3′; human *SYP* reverse primer, 5′-TCA CTC TCG GTC TTG TTG GCA C-3′; human *TUBB3* forward primer, 5′-TCA GCG TCT ACT ACA ACG AGG C-3′, human *TUBB3* reverse primer, 5′-GCC TGA AGA GAT GTC CAA AGG C-3′; human *VIM* forward primer 5′-AGG CAA AGC AGG AGT CCA CTG A-3′, human *VIM* reverse primer 5′-ATC TGG CGT TCC AGG GAC TCA T-3′; human *SMA* forward primer, 5′-GTG GCT ATT CCT TCG TTA CT-3′, human *SMA* reverse primer, 5′-GGC AAC TCG TAA CTC TTC TC-3′; human *CNN* forward primer, 5′-GGT GGA CAT TGG CGT CAA GTA C-3′, human *CNN* reverse primer, 5′-GGG TCA TAG AGA TGC CTT CTC G-3′; human *GFAP* forward primer, 5′-CTG GAG AGG AAG ATT GAG TCG C-3′, human *GFAP* reverse primer, 5′-ACG TCA AGC TCC ACA TGG ACC T-3′; human *PMP22* forward primer, 5′-GCC TTC ATC ACT CCC ACA TT-3′, human *PMP22* reverse primer, 5′-TGA TCG ACA GGA TCA TGG TGG C-3′; human *SERPINF1* forward primer, 5′-TGA AGG CGA AGT CAC CAA GTC C-3′, human *SERPINF1* reverse primer, 5′-CCA TCC TCG TTC CAC TCA AAG C-3′; human *FN* forward primer, 5′-ACA ACA CCG AGG TGA CTG AGA C-3′, human *FN* reverse primer, 5′-GGA CAC AAC GAT GCT TCC TGA G-3′; human *LAMB2* forward primer, 5′-GCG GAC TTG TTC TGA GTG CCA A-3′, human *LAMB2* reverse primer: 5′-ACC TGT GAA GCG GTG ACA CTG A-3′; human *COL4A1* forward primer, 5′-TGT TGA CGG CTT ACC TGG AGA C-3′, human *COL4A1* reverse primer 5′-GGT AGA CCA ACT CCA GGC TCT C-3′; human *IGF-II* forward primer, 5′-TGG CAT CGT TGA GGA GTG CTG T-3′, human *IGF-II* reverse primer 5′-ACG GGG TAT CTG GGG AAG TTG T-3′; human *ACTB* forward primer, 5′-CAC CAT TGG CAA TGA GCG GTT C-3′, human *ACTB* reverse primer, 5′-AGG TCT TTG CGG ATG TCC ACG T-3′. Relative mRNA expression was calculated using the 2^−ΔΔ*CT*^ method normalized over nonsense control, and all experiments were repeated at least three times and presented as mean±s.d.

### Western blotting

Proteins were extracted in radioimmunoprecipitation assay (RIPA) buffer (Cell Signaling Technology) containing a complete protease inhibitor mixture (Roche Molecular Biochemicals, Indianapolis, IN). Protein concentrations were determined by DC protein assay reagent (Bio-Rad) and extracts resolved by SDS/PAGE, then transferred to PVDF membranes (Bio-Rad). Membranes were blocked for 2 h at room temperature in 0.1% TBS-Tween-20 containing 5% non-fat dried milk, washed, and then incubated with the following specific primary antibodies; anti-phospho-Akt (Ser473, #4060 from Cell Signaling Technology, 1:1000 dilution) (Lu et al., 2018), anti-total-Akt (#9272, 1:1000 dilution) (Li et al., 2018), anti-phospho-IGF-I Receptor-β (Tyr1135/1136)/Insulin Receptor-β (Tyr1150/1151) (#3024, 1:500 dilution) (De Filippis et al., 2018), anti-total-IGF1Rβ (sc-713# from Santa Cruz Biotechnology Inc., Dallas, Texas, 1:200 dilution) (Pagesy et al., 2016), anti-phospho-p44/42 ERK1/2 (Thr202/Tyr204, #9101, 1:2000 dilution) (Go et al., 2018), anti-total ERK1/2 (#4695, 1:3000 dilution) (Meng et al., 2018); Anti-Actin (sc-1616 from Santa Cruz Biotechnology, 1:1000 dilution) (Perić et al., 2017); and anti-CgA (HPA017369, Sigma-Aldrich, 1:500 dilution) (Marbiah et al., 2014). After washing, membranes were incubated with HRP-conjugated secondary antibodies (Santa Cruz Biotechnology) and proteins visualized using a Super Signal Chemiluminescence Assay kit (Pierce, Grand Island, NY). The results shown are representative of three independent experiments.

### Tumor Xenograft model

The use of mice was approved by the University of California Los Angeles (UCLA) Animal Research Committee and complied with all relevant federal guidelines and institutional policies. SH-SY5Y shRNA CgA or nonsense control neuroblastoma cells (1×10^6^) in 100 μl matrigel were injected subcutaneously into five-week-old male Nu/J (JAX) mice to generate neuroblastoma tumors (*n*=10 each group). Tumor presence was checked twice weekly. Tumor diameters were measured in two dimensions with Vernier calipers and volumes calculated using the equation length×width^2^×0.5. When the tumor diameter reached 2 cm, mice were euthanized using CO_2_ inhalation. Tumors were excised, weighed and stored at –80C. Tumor tissues (*n*=4 each for SH-SY5Y nonsense- and shRNA CgA-bearing animals) embedded in OCT compound were cut with a cryostat (5 μm) and mounted on commercially available charged slides (Thermo Fisher Scientific). A specific anti-Vimentin antibody (DAKO, M0725, 1:100 dilution) (Hijaz et al., 2016) was used for IHC staining and the percentage of Vimentin immunoreactive cells was calculated by counting cytoplasmic Vimentin positive staining versus total nuclei in ten high power fields. Total RNA from frozen tumor tissue (*n*=2 for SH-SY5Y nonsense-bearing mice and *n*=3 for shRNA CgA-bearing mice) was extracted with RNeasy kit (Qiagen), and reverse transcribed into first-strand cDNA using a cDNA synthesis kit (Invitrogen). Quantitative PCR reactions were carried out to detect tumor tissue *VIM* mRNA expression.

### Statistics

All *in vitro* experiments were repeated at least three times. Results are expressed as mean±s.d. Differences were assessed by Student’s *t*-test using GraphPad Prism 4 (GraphPad Software, La Jolla, CA). *P*-values less than 0.05 were considered significant.
